# Early-onset locally advanced rectal cancer characteristics, a practical nomogram and risk stratification system: a population-based study

**DOI:** 10.3389/fonc.2023.1190327

**Published:** 2023-05-16

**Authors:** Yang Su, Da Shuai Yang, Yan qi Li, Jichao Qin, Lu Liu

**Affiliations:** ^1^ Department of Gastrointestinal Surgery Center, Tongji Hospital, Tongji Medical College, Huazhong University of Science and Technology, Wuhan, China; ^2^ Department of Hepatobiliary Surgery, Renmin Hospital of Wuhan University, Wuhan, China

**Keywords:** early-onset rectal cancer, risk stratification, clinical prediction model, cancer-specific survival, AJCC staging

## Abstract

**Background:**

The purpose of this study is to construct a novel and practical nomogram and risk stratification system to accurately predict cancer-specific survival (CSS) of early-onset locally advanced rectal cancer (EO-LARC) patients.

**Methods:**

A total of 2440 patients diagnosed with EO-LARC between 2010 and 2019 were screened from the Surveillance, Epidemiology, and End Results (SEER) database. The pool of potentially eligible patients was randomly divided into two groups: a training cohort (N=1708) and a validation cohort (N=732). The nomogram was developed and calibrated using various methods, including the coherence index (C-index), receiver operating characteristic curve (ROC), calibration curves, and decision curves (DCA). A new risk classification system was established based on the nomogram. To compare the performance of this nomogram to that of the American Joint Committee on Cancer (AJCC) staging system, DCA, net reclassification index (NRI), and integrated discrimination improvement (IDI) were employed.

**Result:**

Seven variables were included in the model. The area under the ROC curve (AUC) for the training cohort was 0.766, 0.736, and 0.731 at 3, 6, and 9 years, respectively. Calibration plots displayed good consistency between actual observations and the nomogram’s predictions. The DCA curve further demonstrated the validity of the nomination form in clinical practice. Based on the scores of the nomogram, all patients were divided into a low-risk group, a middle-risk group, and a high-risk group. NRI for the 3-, 6-, and 9-year CSS(training cohort: 0.48, 0.45, 0.52; validation cohort: 0.42, 0.37, 0.37), IDI for the 3-, 6-, and 9-year CSS (training cohort: 0.09, 0.10, 0.11; validation cohort: 0.07, 0.08, 0.08). The Kaplan-Meier curve revealed that the new risk classification system possesses a more extraordinary ability to identify patients in different risk groups than the AJCC staging.

**Conclusion:**

A practical prognostic nomogram and novel risk classification system have been developed to efficiently predict the prognosis of EO-LARC. These tools can serve as a guide to individualize patient treatment and improve clinical decision-making.

## 1 Background

Colorectal cancer is a common malignancy of the gastrointestinal system, with the third-highest global incidence and mortality rate among malignant tumors (1). Due to insidious disease, poor specificity of clinical symptoms, and lack of widely used screening tools, a significant proportion of patients are diagnosed with locally advanced rectal cancer (LARC) at the time of diagnosis (2, 3). LARC has garnered considerable attention from researchers domestically and internationally due to its high risk of recurrence and distant metastasis. Early-onset rectal cancer(EORC), which refers to rectal cancer diagnosis in individuals under 50 years, is still not fully understood in terms of its causes and underlying mechanisms (4). In recent years, investigators have studied the clinical and molecular biological features of EORC and found that it may be a separate disease rather than a subgroup of rectal cancer (5, 6). The prognosis of early-onset locally advanced rectal cancer(EO-LARC)may differ from EORC, and a separate survival analysis of EO-LARC is warranted (7). Radical surgical resection is still one of the main treatments for EO-LARC.

The current National Comprehensive Cancer Network (NCCN) clinical practice guidelines primarily rely on the AJCC TNM staging system for predicting prognosis and guiding treatment of EO-LARC patients (8). However, the TNM staging system still has certain limitations, such as age, gender, histological type, degree of tumor differentiation, serum biomarkers, and treatment-related factors affecting patient prognosis (9). Compared with the traditional TMN staging system or other staging systems, nomograms have demonstrated accurate predictive value for many types of tumors and are widely used in clinical applications (10, 11). However, there is no nomogram model to predict the postoperative survival of EO-LARC patients.

In this study, we investigated the factors that affect the postoperative survival of EO-LARC patients based on a large sample dataset from multiple centers in the SEER database and created a nomogram and a novel risk stratification system based on these data to help clinicians make personalized predictions of patient prognosis and guide clinical decisions.

## 2 Methods

### 2.1 Research data

Clinically relevant data for patients diagnosed with EO-LARC between 2010 and 2019 were extracted from the SEER registry database (2010–2019) using SEER*Stat 8.3.9.2 software. This study meets the requirements of the Declaration of Helsinki, and SEER is a publicly available database. The patients’ records and information included in this study were anonymous before analysis. Therefore, institutional ethics committee approval was not required for this study.

### 2.2 Patient inclusion and exclusion criteria

**Figure 1:**
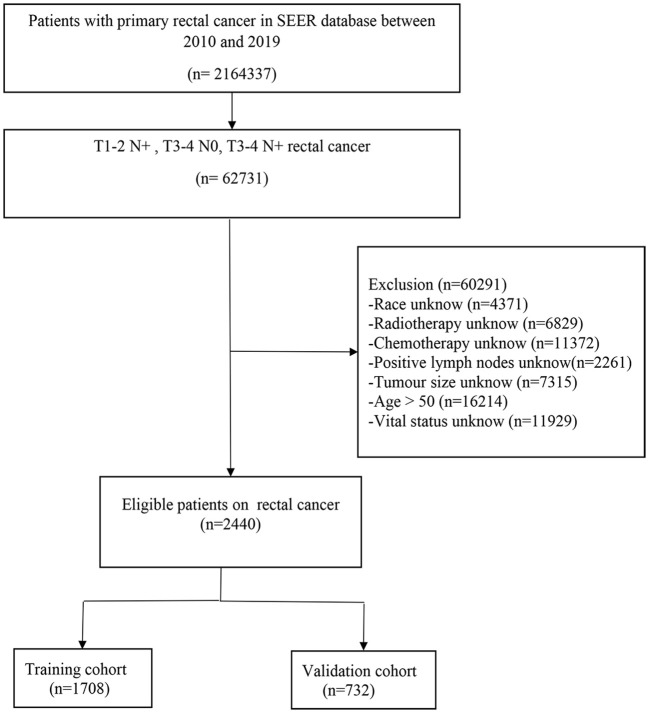
The flowchart of EO-LARC patients identified in the SEER database.

International Classification of Diseases in Oncology (ICD) (C20.9) and ICD code O-3 morphology (8140) were used for differentiation. Inclusion criteria: (a) age <50; (b) confirmed diagnosis of locally progressive rectal cancer (T1-2 N+/T3-4 N0/T3-4 N+); (c) radical surgery; (d) known cause of death. Exclusion criteria: (a) incomplete clinicopathological information; (b) lack of follow-up information; (c) occurrence of distant metastases or undetermined distant metastases; (d) missing treatment options; The process of selection by brush is shown in the flow chart **Figure 1**.

### 2.3 Variables management

Thirteen clinically relevant variables for EO-LARC were downloaded with the seer database, including age, gender, race, pathology, tumor size, T-stage, N-stage, tumor size, lymph node ratio (LNR), CEA, radiotherapy, and survival data were extracted. The main terminal point of the study was the time until cancer-specific death. Tumor staging was performed using the 8^th^ edition AJCC TNM staging criteria.

### 2.4 Establishment of nomogram model

All eligible cases were randomly divided into a training cohort (n=1708, 70% of the total cases) and a validation cohort (n=732, 30% of the total cases). The training cohort was used to build the prognostic model of the nomogram. Meanwhile, the validation cohort was used to test the stability of the model. All variables included in the study were analyzed using univariate Cox regression analysis and multivariate Cox regression analysis to screen for variables that significantly affect postoperative CSS in patients with EO-LARC.

### 2.5 Validation of nomogram model

Based on the training and validation cohorts, the models were validated using C-index, receiver operating characteristics (ROC), calibration curves, and decision curve analysis (DCA). The C-index showed the nomogram’s performance and prediction accuracy, while the ROC showed its sensitivity and specificity. 3-, 6-, and 9-year calibration curves were produced to assess the degree to which model predictions and actual data agreed. The analyses above were done 1,000 times by Bootstrap rerun to lessen bias.

### 2.6 Comparison between risk stratification associated with nomogram and the AJCC staging system

Using the net reclassification index (NRI), C-index, integrated discrimination improvement (IDI), and DCA compared to the AJCC staging system, the nomogram model’s net benefit and risk stratification were evaluated. DCA evaluated the nomogram’s clinical usefulness. Using the best threshold of total score chosen by X-Tile, all eligible patients were separated into low-risk, middle-risk, and high-risk groups. Log-rank tests and Kaplan-Meier curves were used to compare the CSS of patients in various groups.

### 2.7 Statistical methods

All study variables are presented as the number of cases and percentages. Univariate and multi-factor Cox regression analyses, C-index, calibration plots, ROC curves, and DCA curves were generated using R version 3.6.3 and correlation packages. Kaplan-Meier and log-rank tests were applied for survival analysis. Differences in the distributions of the training and validation cohorts were detected by the chi-square test. A two-tailed p-value of less than 0.05 was considered statistically significant.

## 3 Results

### 3.1 Patient characteristics

A total of 2440 patients were diagnosed with early-onset locally advanced rectal cancer and randomized in a 7:3 ratio to the training cohort (1708, 70%) and the validation cohort (732, 30%) (**Figure 1**). The population and clinical features of patients with early-onset locally advanced rectal cancer were summarized below in **Table 1**. Of all patients eligible for inclusion in the research, 1371 (56.19%) were male, 1069 (43.18%) were female, 1910 (78.28%) were white, and 192 (7.87%) were black. Most patients received chemotherapy treatment (90.12%) and radiotherapy (73.98%). The training and validation cohorts were not statistically different in the

**Table 1:** Demographics and clinical and pathology characteristics of EO-LARC patients.

Variable	Whole population		Training cohort		Validation cohort		P value
	n	%	n	%	n	%	
	2440		1708		732		
Age							
17-30	90	3.69%	59	3.45%	31	4.23%	0.35
30-40	565	23.16%	386	22.60%	179	24.45%	
40-50	1785	73.16%	1263	73.95%	522	71.31%	
Race							
Black	192	7.87%	132	7.73%	60	8.20%	0.89
White	1910	78.28%	1341	78.51%	569	77.73%	
Other	338	13.85%	235	13.76%	103	14.07%	
Sex							
F	1069	43.81%	737	43.15%	332	45.36%	0.32
M	1371	56.19%	971	56.85%	400	54.64%	
Pathology							
Adenocarcinoma	2216	90.82%	1546	90.52%	670	91.53%	0.76
Mucinous and signet ring cell carcinoma	127	5.20%	91	5.33%	36	4.92%	
Others	97	3.98%	71	4.16%	26	3.55%	
Grade							
I	159	6.52%	107	6.26%	52	7.10%	0.83
II	1896	77.70%	1329	77.81%	567	77.46%	
III	336	13.77%	236	13.82%	100	13.66%	
IV	49	2.01%	36	2.11%	13	1.78%	
Stages T^a^							
T1	62	2.54%	43	2.52%	19	2.60%	0.90
T2	187	7.66%	135	7.90%	52	7.10%	
T3	1907	78.16%	1334	78.10%	573	78.28%	
T4	284	11.64%	196	11.48%	88	12.02%	
Stages N^a^							
N0	745	30.53%	519	30.39%	226	30.87%	0.73
N1	1174	48.11%	817	47.83%	357	48.77%	
N2	521	21.35%	372	21.78%	149	20.36%	
Tumor size							
0-5	1180	48.36%	838	49.06%	342	46.72%	0.54
5-10	1159	47.50%	799	46.78%	360	49.18%	
>10	101	4.14%	71	4.16%	30	4.10%	
Number							
1	2297	94.14%	1606	94.03%	691	94.40%	0.72
>1	143	5.86%	102	5.97%	41	5.60%	
LNR							
0	1231	50.45%	857	50.18%	374	51.09%	0.58
0-0.2	733	30.04%	509	29.80%	224	30.60%	
0.2-0.4	266	10.90%	196	11.48%	70	9.56%	
>0.4	210	8.61%	146	8.55%	64	8.74%	
CEA							
Positive	955	39.14%	664	38.88%	291	39.75%	0.68
Negative	1485	60.86%	1044	61.12%	441	60.25%	
Radiation							
Yes	1805	73.98%	1271	74.41%	534	72.95%	0.45
No	635	26.02%	437	25.59%	198	27.05%	
Chemotherapy							
Yes	2199	90.12%	1543	90.34%	656	89.62%	0.58
No	241	9.88%	165	9.66%	76	10.38%	

a AJCC (TNM) Stages: The eighth edition AJCC (TNM) staging system.

distribution of the 13 variables (P >0.05).

### 3.2 Analysis of variables

Univariate analysis of the training cohort showed that age, race, sex, pathology, radiotherapy, grade, T-stage, N-stage, CEA, LNR, and radiation were promotional factors for patients with early-onset locally advanced rectal cancer (P<0.05). The findings of multivariate Cox regression analysis showed that sex, pathology, radiotherapy, grade, T-stage, CEA, and LNR were independently prognostic factors affecting CSS in patients with early-onset locally advanced rectal cancer (P<0.05) and were therefore included in the build-up of the nomogram (**Table 2**).

**Table 2:** Univariate and Multivariate Cox regression analyses.

Variable	Univariate		*P* value	Multivariate		*P* value
	HR	95%CI		HR	95%CI	
Age						
17-30	Reference			Reference		
30-40	0.67	0.40-1.10	0.11	0.78	0.47-1.30	0.35
40-50	0.55	0.34-0.88	<0.01	0.71	0.44-1.14	0.16
Race						
Black	Reference			Reference		
White	0.63	0.45-0.90	<0.01	0.67	0.74-1.42	0.13
Other	0.95	0.63-1.43	0.82	1	0.66-1.51	0.87
Sex						
F	Reference			Reference		
M	1.53	1.23-1.90	<0.01	1.45	1.16-1.82	<0.01
Pathology						
Adenocarcinoma	Reference			Reference		
Mucinous and signet ring cell carcinoma	2.09	1.47-2.99	<0.01	1.49	1.03-2.15	<0.01
Others	2.05	1.36-3.09	<0.01	1.47	0.96-2.25	0.07
Grade						
I	Reference			Reference		
II	1.81	0.99-3.32	0.07	1.69	0.92-3.12	0.08
III	4.12	2.19-7.72	<0.01	2.66	1.40-5.05	<0.01
IV	3.51	1.52-8.01	<0.01	2.62	1.12-6.12	<0.01
Stages T^a^						
T1	Reference			Reference		
T2	4.51	2.14-6.38	<0.01	5.37	0.69-3.12	0.10
T3	2.65	1.94-3.71	<0.01	3.14	1.57-4.65	<0.01
T4	5.76	3.61-6.82	<0.01	6.77	4.83-8.15	<0.01
Stages N^a^						
N0	Reference			Reference		
N1	1.53	1.15-2.09	<0.01	1	0.68-1.47	0.98
N2	2.97	2.21-3.99	<0.01	1.01	0.65-1.59	0.93
Tumor size						
0-5	Reference			Reference		
5-10	1.27	1.02-1.57	<0.01	1.08	0.87-1.36	0.45
>10	2.2	1.43-3.38	<0.01	0.98	0.60-1.58	0.94
Number						
1	Reference			Reference		
>1	1.33	0.91-1.95	0.13	1.44	0.95-2.13	0.06
LNR						
0	Reference			Reference		
0-0.20	1.53	1.17-1.99	<0.01	1.75	1.22-2.50	<0.01
0.2-0.4	3.19	2.38-4.28	<0.01	3.28	2.18-4.91	<0.01
>0.4	4.31	3.18-5.84	<0.01	3.57	2.32-5.48	<0.01
CEA						
Positive	Reference			Reference		
Negative	0.52	0.42-0.64	<0.01	0.65	0.52-0.81	<0.01
Radiation						
No	Reference			Reference		
Yes	0.59	0.45-0.78	<0.01	0.56	0.41-0.77	<0.01
Chemotherapy						
Yes	Reference			Reference		
No	0.67	0.44-1.02	0.06	1.32	0.83-2.10	0.23

a AJCC (TNM) Stages: The eighth edition AJCC (TNM) staging system.

### 3.3 Create a nomogram and model validation

**Figure 2:**
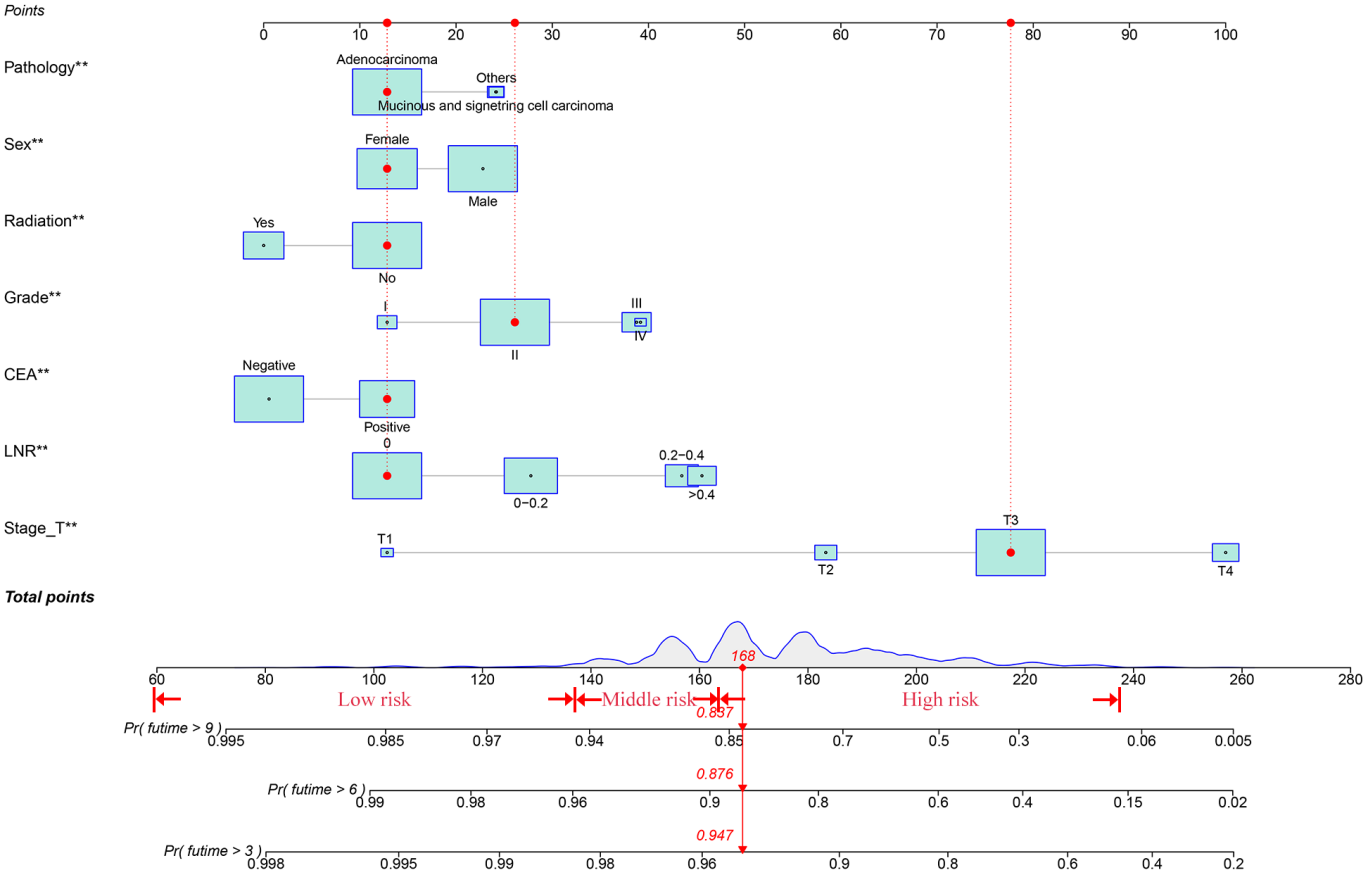
A nomogram for EO-LARC patients and new risk stratification.

**Figure 3:**
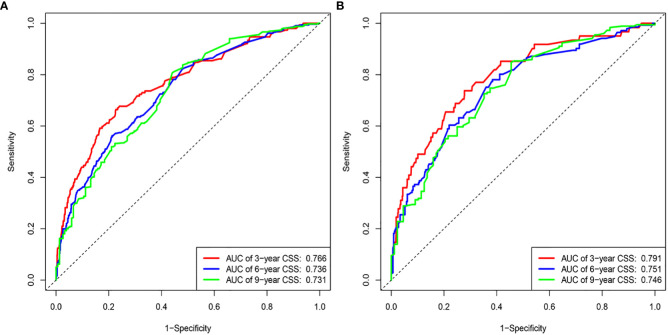
ROC curves. **(A)** Training cohorts based on the nomogram. **(B)** Validation cohorts based on nomogram.

The results of the nomogram, which incorporates all the independent prognostic factors in the multivariate Cox regression model, including sex, pathology, radiotherapy, grade, T-stage, CEA, and LNR, for the prediction of CSS at 3, 6, and 9 years for patients with EO-LARC are shown in **Figure 2**. This nomogram can be used to predict individual CSS according to different clinicopathological characteristics of patients. The internal validation C-index of the model assesses the model’s accuracy; the calibration curve assesses the consistency of the predicted values with the actual survival. The C-indexes for the training and validation cohorts were 0.747 (95% CI:0.735-0.752) and 0.744 (95% CI: 0.731-0.756), respectively (**Figure 3**). The ROC curves, DCA curves, and calibration curves are shown in **Figures 3** **–** **5**. The results of the ROC curve analysis showed that the AUCs for the training cohort at 3, 6, and 9 years were 0.766, 0.736, and 0.731, respectively. The AUCs for the validation cohort at 3, 6, and 9 years was 0.791, 0.751, and 0.746, respectively. The calibration curves all showed that the 3, 6, and 9-year predicted CSS probabilities strongly agreed with the actual observations. In addition, the DCA curves at 3, 6, and 9 years showed outstanding positive clinical net benefits in both the training and validation cohorts. e validation cohort. DCA, decision curve analysis; CSS, cancer-specific survival.

**Figure 4:**
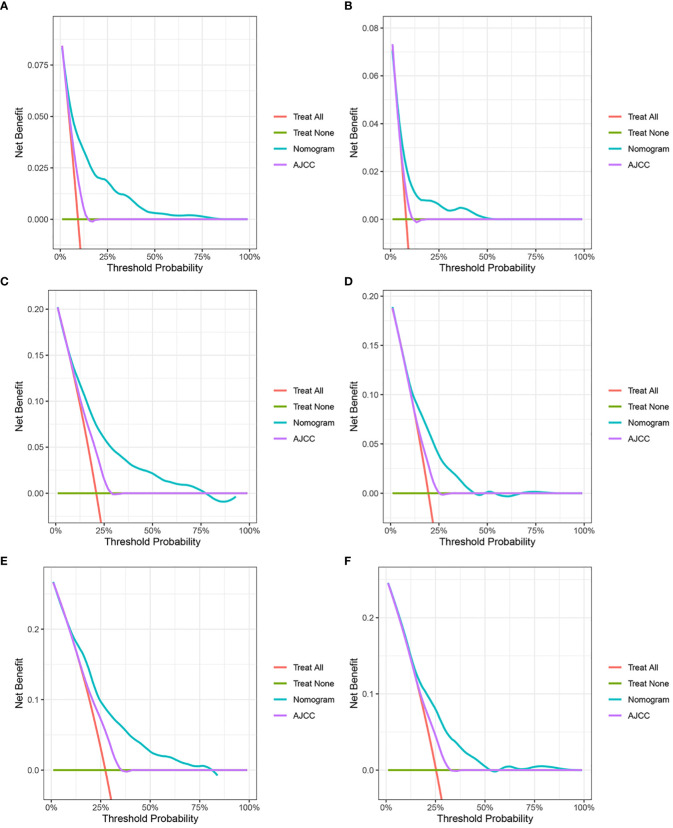
Decision curve analysis. **(A, C, E)** DCA curves of 3-year, 6-year, and 9-year CSS in the training cohort. **(B, D, F)** DCA curves of 3-year, 6-year, and 9-year CSS in the validation cohort. DCA, decision curve analysis; CSS, cancer-specific survival.

**Figure 5:**
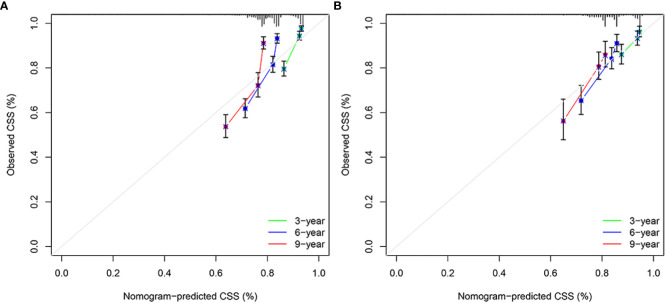
Calibration plots of 3-year, 6-year, and 9-year CSS for EO-LARC patients. **(A)** Calibration plots of 3-year, 6-year, and 9-year CSS in the training cohort. **(B)** Calibration plots of 3-year, 6-year, and 9-year CSS in the validation cohort. CSS, cancer-specific survival.

### 3.4 Comparison of the new model with the traditional pTNM model

**Figure 6:**
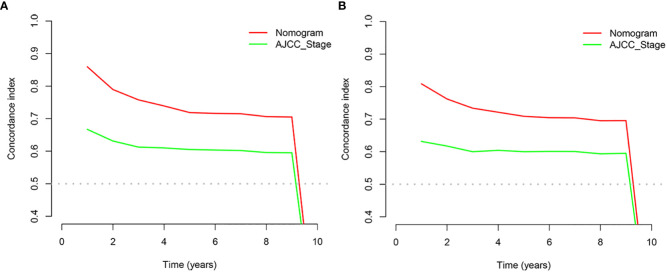
C-index analysis. **(A)** Nomogram-related C-index and AJCC staging criteria-related C-index in the training cohort. **(B)** Nomogram-related C-index and AJCC staging criteria-related C-index in the validation cohort.

In the training and validation cohort, the C-index of the nomogram was all higher than that of the AJCC staging system (**Figure 6**). The 3-, 6-, and 9-year NRIs were 0.48 (95% CI=0.40-0.65), 0.45 (95% CI=0.36-0.66), and 0.52 (95% CI=0.37-0.71), respectively (**Table 3**). IDI (training cohort: 3-, 6-, 9-year CSS: 0.09, 0.10, 0.11; validation cohort: 3-, 6-, 9-year CSS: 0.07, 0.08, 0.08) indicated that the established nomogram significantly outperformed AJCC TNM staging system (P<0.05) (**Table 3**). The net benefit of the nomogram was compared to that of the AJCC staging system. The DCA curves showed that the nomogram had a higher net benefit and clinical validity than the 8th edition of the AJCC TNM staging system in the training and validation cohorts (**Figure 4**).

**Table 3:** The nomogram and AJCC staging criteria for NRI and IDI are in the CSS projections for EO-LARC.

Index	Training cohort		*P* value	Validation cohort		*P* value
	Value	95%CI		Value	95%CI	
NRI						
3-year CSS	0.48	0.40-0.65		0.42	0.34-0.54	
6-year CSS	0.45	0.36-0.66		0.37	0.26-064	
9-year CSS	0.52	0.37-0.71		0.37	0.20-0.69	
IDI						
3-year CSS	0.09	0.06-0.13	<0.001	0.07	0.05-0.11	<0.001
6-year CSS	0.10	0.07-0.14	<0.001	0.08	0.06-0.13	<0.001
9-year CSS	0.11	0.07-0.15	<0.001	0.08	0.07-0.14	<0.001

### 3.5 Risk stratification based on the nomogram

**Figure 7:**
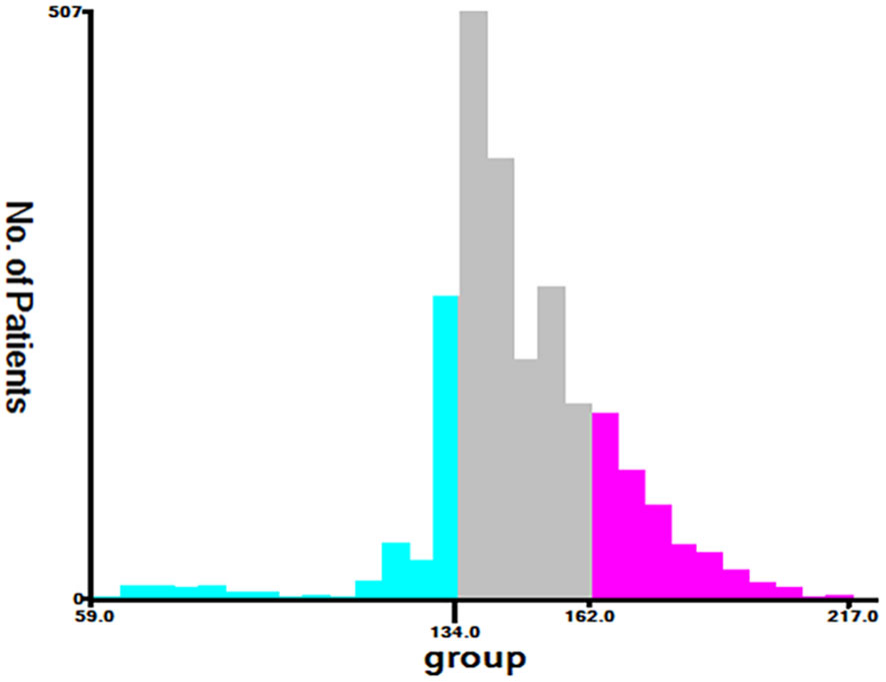
Cut-off point for risk stratification selected using X-tile.

**Figure 8:**
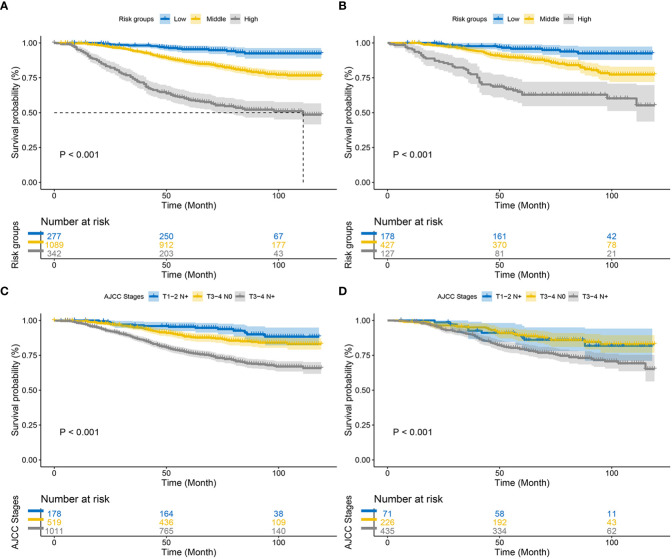
Kaplan–Meier CSS curves of patients with EO-LARC based on different criteria. **(A, B)** Kaplan–Meier CSS curves of the training and validation cohorts based on the new risk stratification system. **(C, D)** Kaplan–Meier CSS curves of the training and validation cohorts based on AJCC staging criteria.

Finally, using the total points determined by the nomogram, we created a risk stratification system. Three risk groups of EO-LARC patients were created: low risk (total points < 134), middle risk (134 ≤ total points <162), and high risk (total points ≥ 162). (**Figure 7**). The AJCC staging approach had a limited ability to identify high-risk patients in both the training and validation cohorts, but the Kaplan-Meier CSS curves demonstrated excellent differentiation among the three risk groups (**Figure 8**).

## 4 Discussion

The incidence of EORC patients is increasing annually, and the results of their survival analysis have been reported successively (12, 13). Accurate prediction of patient survival prognosis can assist medical personnel in making individualized treatment and follow-up decisions. While the AJCC TNM staging system is currently the most commonly used prognostic assessment system, relying solely on anatomic invasion and metastasis of tumors may impact the accuracy of survival prediction. In recent years, several clinical prediction models for predicting tumor prognosis have emerged and shown the superior predictive ability of the AJCC TNM staging system (14, 15). Clinical prediction models for projecting EO-LARC patients are relatively rare.

In this study, based on univariate and multifactorial COX proportional risk regression analysis, sex, pathology, radiotherapy, grade, T-stage, CEA, and LNR were independent risk factors affecting the postoperative outcome of EO-LARC patients. Gender differences had a significant effect on postoperative EO-LARC. This study found a worse prognosis in male patients (HR=1.45; 95% CI=1.16-1.82; P<0.01), which aligns with most of the literature. The protective effect of estrogen and differences in pregnancy, birth, anatomy, and physiology may be associated with a relatively lower incidence and better prognosis of CRC in women (16).

Mucinous and signet ring cell carcinoma were identified as independent risk factors for postoperative EO-LARC patients (HR=1.49; 95% CI=1.03-2.15; P<0.01), consistent with many recent studies on the relationship between tumor type and prognosis (17). Signet ring carcinoma is a rare type of colorectal cancer with a low incidence. It belongs to a particular kind of invasive adenocarcinoma along with mucinous adenocarcinoma, which has a high incidence of tumor infiltration depth, lymph node metastasis, vascular tumor embolism, and combined intestinal obstruction, with a late stage of disease at the time of patient presentation, a low surgical resection rate, and a poor prognosis.

Radiotherapy is an essential tool in the treatment of EO-LARC, and the European Society for Medical Oncology (ESMO) guidelines recommend two preoperative radiotherapy modalities: long-course radiotherapy combined with concurrent chemotherapy. Still, short-course preoperative radiotherapy is the predominant treatment in some European countries (18). This study showed that patients could benefit from radiotherapy (HR=0.56; 95% CI=0.41-0.77; P<0.01). The effect of tumor differentiation on the postoperative outcome of colorectal cancer patients has been studied more frequently, and the lower the degree of tumor cell differentiation tends to be more malignant, less sensitive to treatment such as radiotherapy, and less favorable overall treatment prognosis. The College of American Pathologists used the degree of tumor differentiation as a class IIA prognostic factor for colorectal cancer (19). The results of the present study also showed that high-grade (low/undifferentiated) was an independent risk factor for postoperative CSS in patients with EO-LARC (P<0.001), and the degree of differentiation was significant for the prognosis of rectal cancer.

This study found that T-stage was an independent risk factor for postoperative CSS in EO-LARC patients by multifactorial analysis (P<0.01). That is, the more extensive invasion of the primary focus within a specific range, the more involvement of nearby lymph nodes, or the more distant metastasis of the tumor, the worse the prognosis of patients. A study on colon cancer noted that the 5-year survival rate of patients with high T-stage was much lower than that of patients with T1-stage (20).

Carcinoembryonic antigen (CEA) is a standard tumor marker in colorectal cancer. It can, to some extent, provide a basis for tumor diagnosis, recurrence, and metastasis and is most effective when patients have high preoperative serum CEA levels (21). The results of this study are consistent with previous studies (22, 23), where elevated preoperative CEA was an independent risk factor for postoperative EO-LARC patients (HR=1.24; 95% CI=1.05-1.47; P<0.001), which is also consistent with clinical reality.

Lymph node metastasis is a common form of metastasis in colorectal cancer, which can lead to disease recurrence and even death. The rate of lymph node metastasis is calculated by dividing the number of metastatic lymph nodes by the number of pathologically examined lymph nodes. Compared with the traditional number of lymph node metastases, it can effectively avoid differences due to individual patient factors. It can be used for lymph node staging and prognosis assessment of colorectal patients (24, 25). The study results indicated that lymph node metastasis rate (LNR) (HR=1.24; 95% CI=1.05-1.47; P<0.001) had a high predictive value for the survival of EO-LARC patients.

Nomograms are an intuitive and easy-to-understand statistical tool that can consider multiple risk factors and provide individualized assessments of patients. This study conducted a multifactorial survival analysis, which included seven objective clinical and pathological factors (sex, pathology, radiotherapy, grade, T-stage, CEA, and LNR) to construct a nomogram that predicts CSS at 3, 6, and 9 years in patients with EO-LARC. The C-index, NRI, ROC, and IDI demonstrated that the nomogram had better clinical value than AJCC staging. Furthermore, EO-LARC patients were classified into low, medium, and high-risk groups based on the total score of the nomogram. The results of Kaplan-Meier and Cox risk ratio models, showed significant differences in CSS between these three groups.

Although the EO-LARC patients included in this study were rigorously screened, several limitations remain: (i) The SEER database does not contain detailed treatment protocols, gene expression information, immunotherapy, and other indicators, which may affect the accuracy and comprehensiveness of the prediction model. (ii) Retrospective studies may lead to inherent bias, and direct deletion of patients with missing data may introduce selection bias. (iii) The lack of independent external validation in the study may affect the practical generalizability of the prediction model. The selection of predictors still needs to be optimized in the future and confirmed based on prospective randomized clinical trials.

## 5 Conclusion

In conclusion, this study constructed and validated a prognostic nomogram, which provides a simple and reliable tool for survival prediction of EO-LARC patients after surgery. Meanwhile, the new risk stratification model can conveniently screen patients with different risks, which is important for the individualized treatment of EO-LARC cancer patients.

## Data availability statement

The datasets presented in this study can be found in online repositories. The names of the repository/repositories and accession number(s) can be found in the article/supplementary material.

## Ethics statement

All methods have adhered to the relevant official SEER database guidelines and regulations.

## Author contributions

Conceptualization: YS. Data curation: DY. Formal analysis: YS and YL. Writing-original draft: YS. Writing-review and editing: LL and JQ. All authors contributed to the article and approved the submitted version.
